# The debut of PMC Biophysics

**DOI:** 10.1186/1757-5036-1-1

**Published:** 2008-11-05

**Authors:** Huan-Xiang Zhou

**Affiliations:** 1 Editor-in-Chief, *PMC Biophysics*, Department of Physics and Institute of Molecular Biophysics, Florida State University, Tallahassee, FL 32306, USA

Welcome to PMC Biophysics, an international, peer-reviewed, open access journal devoted to biological physics in all its myriad forms. The journal has been launched in response to two significant trends in publishing and research.

Firstly, the open access movement is gathering strength. Pioneered by PMC Biophysics's parent company, BioMed Central, open access allows any reader immediate free access to published articles, encouraging the widest possible spread of knowledge. Archiving in multiple repositories ensures the long-term preservation of scientific research, and electronic publication enables the inclusion of multimedia content and the dissemination of raw data - allowing other researchers to exploit them. All these features serve to enhance the impact of published articles.

The strength and future success of open access are heralded by two major recent developments. The US National Institutes of Health, the largest public funding agency for biomedical research, now requires that all peer-reviewed journal articles arising from NIH funding be made open access within 12 months of publication. In addition, BioMed Central has recently been acquired by Springer, the world's second largest publisher of scientific journals.

The second trend concerns the multidisciplinary nature of research in biological physics.

Over the past 40 years, the field of biological physics has been shaped by forces from two directions. On one hand, the study of biological systems has become less and less descriptive and more and more quantitative. It has become more compelling to model biological systems and rationalise or predict behaviours from physical principles, such as in protein folding.

On the other hand, it has been discovered time and again that models and techniques developed for simple physical systems eventually become powerful tools for studying biological systems – Born's model for ion solvation and Kramers' rate theory for barrier crossing being classic examples. X-ray crystallography and computer simulations both started from humble beginnings, but are now used to study ever larger biomacromolecular complexes. The end result is that biological physics is now a thriving area for researchers from a wide variety of backgrounds, united by their interest in, or their work's application to, biological systems.

With the help of the distinguished members of our editorial board and our colleagues who willingly serve as reviewers, I hope that PMC Biophysics will become a powerful voice in biological physics, and your first choice when considering venues for publishing your work.

